# Associations of *CYP2B6* genetic polymorphisms with Hirschsprung’s disease in a southern Chinese population

**DOI:** 10.1002/jcla.24074

**Published:** 2021-11-09

**Authors:** Yanqing Liu, Chaoting Lan, Bingxiao Li, Ning Wang, Xiaoyu Zuo, Lihua Huang, Yuxin Wu, Yun Zhu

**Affiliations:** ^1^ Department of Pediatric Surgery Guangzhou Institute of Pediatrics Guangzhou Women and Children’s Medical Center Guangzhou Medical University Guangzhou China; ^2^ The First Affiliated Hospital of Jinan University Guangzhou China; ^3^ Guangzhou Medical University Guangzhou China

**Keywords:** CYP2B6, cytochrome P450 family 2 subfamily B member 6, Hirschsprung's disease, HSCR, single‐nucleotide polymorphism

## Abstract

**Background:**

Hirschsprung’s disease (HSCR) is an enteric nervous system birth defect partially caused by a genetic disorder. Single‐nucleotide polymorphisms (SNPs) of the cytochrome P450 family 2 subfamily B member 6 (CYP2B6) gene are reported to be associated with HSCR.

**Methods:**

We evaluated the association of rs2054675, rs707265, and rs1042389 with HSCR susceptibility in southern Chinese children including 1470 HSCR patients and 1473 controls using the TaqMan SNP Genotyping Assay.

**Results:**

rs2054675 C allele and the rs707265 G allele were risk SNPs for total colonic aganglionosis (OR = 1.82, 95% CI 1.29 ~ 2.55, *P*_*adj* < 0.001 and OR = 0.68, 95% CI 0.48 ~ 0.97, *P*_*adj* = 0.034). These results suggested that CYP2B6 rs2054675 and rs707265 polymorphisms were associated with increased susceptibility to the severe HSCR subtype in southern Chinese children.

**Conclusion:**

We suggest that CYP2B6 rs2054675 and rs707265 polymorphisms are associated with increased susceptibility to the severe HSCR subtype in southern Chinese children.

## Graphical abstract

Hirschsprung's disease (HSCR) is an enteric nervous system birth defect partially caused by a genetic disorder. Single‐nucleotide polymorphisms (SNPs) of the cytochrome P450 family 2 subfamily B member 6 (CYP2B6) gene are reported to be associated with HSCR. We evaluated the association of rs2054675, rs707265 and rs1042389 with HSCR susceptibility in southern Chinese children including 1470 HSCR patients and 1473 controls. rs2054675 C allele and the rs707265 G allele were risk SNPs for total colonic aganglionosis (TCA) (OR = 1.82, 95% CI 1.29 ~ 2.55, P_adj <0.001 and OR = 0.68, 95% CI 0.48 ~ 0.97, P_adj = 0.034). These results suggested that CYP2B6 rs2054675 and rs707265 polymorphisms were associated with increased susceptibility to the severe HSCR subtype in southern Chinese children.

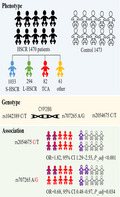

## 1 INTRODUCTION

Hirschsprung's disease (HSCR) is a congenital gastrointestinal (GI) disease in which submucosal and intramuscular plexus ganglion cells are lacking in the intestinal wall of the distal digestive tract, which is caused by developmental disorders of the enteric nervous system during embryonic development.^1^ The occurrence of HSCR shows sex‐related and racial disparities, with a male‐to‐female ratio of 4/1 and a higher incidence in Asia, including China (1/3500 vs. 1/5000).^2^ Hirschsprung's disease can be divided into 3 subtypes depending on the length of the aganglionic tract, including short‐segment HSCR (S‐HSCR), long‐segment HSCR (L‐HSCR), and total colonic aganglionosis (TCA), with the rare occurrence of cumulative full‐bowel megacolon.^3^ In addition, according to the presence or absence of other malformations and chromosomal abnormalities, HSCR can be divided into simple and syndrome types, and the most common comorbidity is trisomy 21.^4^ Moreover, there are familial and sporadic types of HSCR according to family inheritance. More than one‐fifth of HSCR cases show familial aggregation, whereas most HSCR cases are sporadic.^5^

The pathogenesis of HSCR is complex and involves multiple genes and multiple signaling pathways. A genome‐wide association study (GWAS) has identified a variety of genes associated with a high risk of HSCR in the RET and EDNRB signaling pathways, which play important roles in the migration of enteric neuron crest cells during the development of the enteric nervous system (ENS).^6^ RET intronic enhancer rs2435357 T allele, rs2506004 C>A were associated with a 4‐fold increase in risk of HSCR. These variants can alter the binding of transcriptional factors (SOX10, ARNT5/NXF, and HOXB5) and decreased the expression of RET gene.^7^ Other common variants of SEMA3C, SEMA3D, NRG1, 19q12, 3p21, VAMP5, and MCC are widely reported to be independently or synergistically associated with the risk of HSCR.^8^, ^9^ However, only a small proportion of HSCR cases can be explained by these known factors; for most sporadic cases, missing heritability remains to be identified. Therefore, it is believed that interaction between environmental and genetic factors plays a crucial role in the pathogenesis of HSCR,^10^ which remains barely explored.

The human *CYP2B6* gene belongs to the human cytochrome P450 enzyme system (CYPs), which are involved in the metabolism of fatty acids, cholesterol, bile acids, vitamin D, retinoids, and eicosanoids.^11^ They play a role in some diseases occurring during embryogenesis and infantile development.^12^ Notably, P450 family members are environmental responders to dietary components, chemical inducers and signals (i.e., pheromones), drugs, etc.^13^ These three SNPs (rs707265, rs1042389, and rs2054675) which were likely to be regulatory variants and satisfied the criteria regarding the minor allele frequency, Hardy–Weinberg equilibrium (HWE) and linkage disequilibrium, were validated using a cohort (262 cases and 290 controls) from eastern Chinese population.^14^ Considering distinctive dietary and toxic or drug exposure between eastern and southern Chinese population, we conducted an association study in a southern Chinese population (1470 cases and 1473 controls) to evaluate the association between *CYP2B6* polymorphism and susceptibility to HSCR. This study will provide a hint of environmental factors for pathogenesis of HSCR.

## 2 MATERIALS AND METHODS

### 2.1 Study subjects

1740 HSCR patients and 1473 controls from southern China were recruited from Guangzhou Women and Children's Medical Center as described previously,^8^ and their detailed clinical information is summarized in Table 1. The diagnosis of HSCR was confirmed in all cases by pathological biopsies of intestinal tissue obtained from surgery showing a lack of submucosal and intramuscular plexus ganglion cells. The patients with HSCR were divided into the 3 subtypes of S‐HSCR, L‐HSCR, and TCA based on the length of aganglionosis in the pathological biopsy. Control samples from individuals without HSCR or neurological disease were randomly selected. The study was ethically approved by the Institutional Review Board of Guangzhou Women and Children's Medical Center, and informed consent was obtained from the guardians of all subjects in this study (Ethical Approval Number: 201943800).

**TABLE 1:** Clinical Characteristics of the Study Population

Characteristic	Cases (*n* = 1470)	Controls (*n* = 1473)	*P^a^*
Sex (Male; Female)	1230/240	1015/458	<0.001
Age (month)^b^ (≤2;>2)	8.37 ± 20.50	18.61 ± 19.75	<0.001
S‐HSCR^c^ (%)	1033 (70.3%)	N/A	–
L‐HSCR^c^ (%)	294 (20.1%)	N/A	–
TCA^c^ (%)	82 (5.6%)	N/A	–
TIA^c^ (%)	3 (0.2%)	N/A	–
Unknown subtype	58 (0.7%)	N/A	–
Syndromic HSCR (%)	48 (3.3%)	N/A	–
With Constipation	162 (11.0%)	N/A	–
Presurgery Enteritis (%)	261 (17.8%)	N/A	–
Postsurgery Enteritis (%)	249 (16.9%)	N/A	–

a. Two‐tailed χ2 test of the distribution between HSCR cases and controls.

b. Age (month) of onset for HSCR cases: (mean ± SD). SD, Standard deviation; NA, Not available.

c. S‐HSCR, short‐segment HSCR; L‐HSCR, Long‐segment HSCR; TCA, Total colonic aganglionosis; TIA, Total intestine aganglionosis.

### 2.2 SNP selection and genotyping

*CYP2B6* rs707265, rs1042389, and rs2054675 were selected using criteria as described in our previous study.^15^ Briefly, the candidate SNPs that were likely to be regulatory variants and satisfied the criteria regarding the minor allele frequency, Hardy–Weinberg equilibrium (HWE) and linkage disequilibrium were selected for validation.

TIANamp Blood Genomic DNA Kits and TIANquick FFPE DNA Kits (TIANGEN Biotech Co. Ltd.,) were applied to isolate genomic DNA from venous blood and paraffin samples. Subsequently, *CYP2B6* SNPs were genotyped using the TaqMan SNP Genotyping Assay on an ABI‐7900 real‐time quantitative PCR instrument (Applied Biosystem).^16^ Three replicates were performed for each sample.

### 2.3 Correlation analysis of genotype and gene expression

The associations between 3 SNPs (rs2054675, rs707265, and rs1042389) and *CYP2B6* gene expression in colon tissues, or the expression quantitative trait locus (eQTL) effect, were evaluated through the GTEx Portal database (https://www. gtexportal.org/home/). We used “Single‐Tissue eQTLs” and “Single‐Tissue sQTLs”, and only chose colon tissues to show eQTLs or sQTLs results of CYP2B6 rs707265, rs1042389, and rs2054675. These results were drawn using “eQTL violin plot” provided by GTEx Portal database, which showed the median, quartiles, and outliers as well as eQTL P‐value.

### 2.4 Statistical analysis

The statistical analysis of all data in this study was performed by using SAS (version 9.4; SAS Institute). The differences in age or sex between the HSCR and control groups were compared using a two‐tailed chi‐square test. The Hardy–Weinberg equilibrium test was performed in the control group to assess genotyping quality, and *p* > 0.05 was considered to indicate a satisfactory goodness‐of‐fit. Multiple logistic regression analysis was applied to assess the association of *CYP2B6* polymorphisms with the risk of HSCR as well as HSCR subtypes (S‐HSCR, L‐HSCR, and TCA). *P_crude* and *P_adj* indicate the association significance without or with adjusting the effects of age and sex. Odds ratios (ORs) were compared between the HSCR and control groups.

## 3 RESULTS

### 3.1 eQTL analysis

**FIGURE 1:**
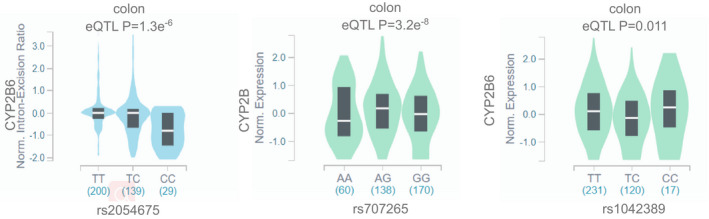
Associations of rs2054675, rs707265 and rs1042389 genotypes with *CYP2B6* mRNA splicing or expression in colon tissues based on data from the GTEx portal database (https://www.gtexportal.org/home/). The boxplot represents the *CYP2B6* intro‐excision ratio or mRNA expression according to the rs2054675, rs707265 and rs1042389 genotypes

To investigate the functional potential of the SNPs, we used the Genotypic Tissue Expression (GTEx) dataset to evaluate the associations of rs2054675, rs707265, and rs1042389 with *CYP2B6* expression.^17^ We found that rs2054675 and rs707265 were significant splicing quantitative trait loci (eQTLs) (*p* = 1.3e^−6^) and expression quantitative trait loci (eQTLs) (*p* = 3.2e^−8^) of the *CYP2B6* gene in colon tissues, but rs1042389 was not significant (Figure 1). The results verified regulatory potential of rs2054675 and rs707265.

### 3.2 Association of *CYP2B6* SNPs with HSCR susceptibility

Subsequently, the genotype frequencies of rs2054675, rs707265, and rs1042389 and their associations with HSCR were calculated and are summarized in Table 2. The genotypes of the 3 SNPs in the control group were in HWE (*p* > 0.05). To better illustrate the pattern of the effects of rs2054675, rs707265, and rs1042389 on *CYP2B6*, an association test was applied by logistic regression under four different genetic models (allelic genetic, genotypic, dominant, and recessive models). The results showed that the three SNPs did not present a significant association with HSCR in general. Association of rs1042389 under the dominant model was close to significance (*P_adj* = 0.060, OR = 1.14, 95% CI of 0.98 ~ 1.32) (Table 2).

**TABLE 2:** Replication results for three selected SNPs in a southern Chinese population of 1470 cases and 1473 controls

CHR	SNP	BP	Gene	A1/A2	Model	Patient	Control	OR (CI 0.95)	*P_crude*	*P_adj*
19	rs2054675	40989850	*CYP2B6 upstream*	C/T	ALLELIC	670/2160	697/2225	0.99 (0.88 ~ 1.12)	0.871	0.975
					GENO	76/518/821	82/533/846	–	0.965	0.122
					DOM	594/821	615/846	1.00 (0.86 ~ 1.15)	0.954	0.181
					REC	76/1339	82/1379	0.95 (0.69 ~ 1.32)	0.787	0.956
19	rs707265	41018182	*CYP2B6 3'UTR*	A/G	ALLELIC	874/1974	944/1960	0.92 (0.82 ~ 1.03)	0.145	0.161
					GENO	131/612/681	165/614/673	–	0.164	0.212
					DOM	743/681	779/673	0.94 (0.81 ~ 1.09)	0.432	0.883
					REC	131/1293	165/1287	0.79 (0.62 ~ 1.01)	**0.057**	0.373
19	rs1042389	41018248	*CYP2B6 3'UTR*	C/T	ALLELIC	995/1855	966/1954	1.08 (0.97 ~ 1.21)	0.144	0.088
					GENO	165/665/595	163/640/657	–	0.213	0.823
					DOM	830/595	803/657	1.14 (0.98 ~ 1.32)	0.079	**0.060**
					REC	165/1260	163/1297	1.04 (0.83 ~ 1.31)	0.734	0.885

Abbreviations: A1/A2, Minor allele/major allele; BP, Base pair where the SNP is located; CHR, Chromosome; *CYP2B6*, Cytochrome P450 2B6; Freq, Risk allele frequency of the SNP in cases or controls. ALLELIC, GENO, DOM, and REC, association tests following allelic genetic, genotypic, dominant and recessive models. The calculation of odds ratio (OR) is also based on the risk allele of each SNP; Gene.refgene, The gene where the SNP located; P_adj, P value adjusted by sex; P_crude, Association test by logistic regression; SNP, Single‐nucleotide polymorphism.

### 3.3 Stratification analysis of *CYP2B6* SNPs with HSCR subtypes

Considering the effects of HSCR subtypes in the population, a stratification analysis of the three SNPs with HSCR subtypes was performed. There were 3 common subtypes of HSCR with increasing severity: S‐HSCR, L‐HSCR, and TCA. We analyzed the associations of rs2054675, rs707265, and rs1042389 and the three HSCR subtypes. The results indicated that TCA was significantly associated with rs2054675 (*P_adj* <0.001, OR = 1.82, 95% CI of 1.29 ~ 2.55) and rs707265 (*P_adj* = 0.034, OR = 0.68, 95% CI of 0.48 ~ 0.97), although there was no significant association between rs1042389 and any subtype (Table 3).

**TABLE 3:** Association results for three independent SNPs related to different subclinical features classified by aganglionosis length, including short‐length (S‐HSCR), long‐length (L‐HSCR) and TCA

CHR	SNP	BP	A1/A2	Aganglionic status	Patient	Control	OR (CI 0.95)	*P_crude*	*P_adj*
19	rs2054675	40989850	C/T	S‐HCSR	464/1534	697/2225	0.97 (0.85 ~ 1.12)	0.610	0.701
				L‐HSCR	130/436		0.95 (0.77 ~ 1.18)	0.652	0.671
				TCA	57/101		1.82 (1.29 ~ 2.55)	**<0.001**	**<0.001**
19	rs707265	41018182	A/G	S‐HCSR	614/1388	944/1960	0.91 (0.80 ~ 1.03)	0.182	0.147
				L‐HSCR	180/388		0.96 (0.79 ~ 1.16)	0.713	0.665
				TCA	42/128		0.68 (0.48 ~ 0.97)	**0.039**	**0.034**
19	rs1042389	41018248	C/T	S‐HCSR	708/1304	966/1954	1.09 (0.97 ~ 1.24)	0.133	0.162
				L‐HSCR	201/365		1.12 (0.92 ~ 1.35)	0.261	0.265
				TCA	50/112		0.90 (0.64 ~ 1.27)	0.562	0.550

Abbreviations: A1/A2, Risk allele and protective allele for the disease; BP, Base pair where the SNP is located; CHR, Chromosome; *CYP2B6*, Cytochrome P450 2B6; Freq, Risk allele frequency of the SNP in cases or controls. The calculation of the odds ratio (OR) is also based on the risk allele of each SNP; Func.refgene, Functional role of the SNP in the gene; Gene.refgene, Gene where the SNP is located; P_adj, P value adjusted by sex; P_crude, Association test by logistic regression; SNP, Single‐nucleotide polymorphism.

## 4 DISCUSSION

The human cytochrome P450 enzyme system (CYP) has been reported to mediate some diseases of neonatal development. Type 3 mutations of CYP7B1 result in congenital bile acid defects, whereas type 5A mutations result in autosomal recessive hereditary spastic paraplegia, due to the disruption of cholesterol homeostasis in the liver and upper motor neurons.^18^ CYP24A1 deficiency was recently shown to lead to severe infantile hypercalcemia.^19^ CYP11A1 localizes to mitochondria and produces steroid hormones, and deficiency of these hormones causes congenital lipoid adrenal hyperplasia.^20^ CYP1B1 plays an important role in the embryonic development of the eyes. CYP1B1 mutations cause primary congenital glaucoma.^21^ CYP2B6 is one of the most important exogenous toxic metabolic enzymes in the CYP family. *CYP2B6* is mainly distributed in the human liver, GI tract, pancreas, kidney, and reproductive system and participates in the synthesis and metabolism of a variety of endogenous and exogenous substances.^22^, ^23^ The substantial expression of *CYP2B6* in the GI tract indicates a potential role in enteric diseases. CYP2B6 gene polymorphism is related to the occurrence of bronchopulmonary dysplasia (BPD) (especially in infants with extremely low birth weight),^24^ acute myeloid leukemia (AML),^25^ and breast cancer.^26^ Reduced activity of *CYP2B6* enzyme due to gene polymorphism is associated with increased response to drug sensitivity and infection.^27^, ^28^, ^29^, ^30^

P450 family members, encoding exogenous toxic metabolic enzymes, are environmental responders to dietary components, chemical inducers and signals (i.e., pheromones), drugs, etc.,^13^ the activity of which varied in different ethnicities.^31^ CYP2B6 gene polymorphism can explain different effects of antiviral drugs in a specific population.^32^, ^33^ Three SNPs of *CYP2B6* (rs707265, rs1042389, and rs2054675) validated in this study were reported to influence drug metabolization. rs1042389C was associated with low Efavirenz(anti‐HIV drug) response in South African black population through affecting the binding of miRNAs at 3’UTR.^34^, ^35^ rs707265 had effects on methadone metabolism and pharmacodynamics, whereas rs2054675 affected pharmacokinetics of an antidiabetic drug pioglitazone. For the association with HSCR, this study showed different results in southern Chinese population from eastern Chinese population. rs707265 was associated with HSCR susceptibility in eastern Chinese population, but none of the three SNPs were significantly associated with HSCR subtype.^14^ However, in this study, the association of CYP2B6 SNPs with HSCR was not validated, which might reflect different toxic factors from the environment between eastern and southern Chinese population.

Additionally, we found that CYP2B6 rs2054675 and rs707265 polymorphisms are associated with increased susceptibility to the severe HSCR subtype(TCA) in southern Chinese children. TCA is the most severe and rare subtype of HSCR with 1% incidence rate. Previous studies showed that genetic factors were supposed to contribute more to the pathogenesis of TCA than other subtypes,^36^ which might explain the significant association between rs2054675C and rs707265 G alleles with TCA risk, but not with other subtypes. Few TCA patients and different toxic or drug exposure between eastern and southern Chinese population might explain a nonsignificant association with TCA in the previous study. To increase statistical power, we should extend these 150 TCA patients in this study to a big cohort. With more TCA patients recruited in this cohort, toxic or drug exposure should be considered in the association analysis. Further investigation of the role of *CYP2B6* in HSCR will facilitate the elucidation of the mechanism whereby environmental factors (including diet and drugs) affect birth defects in TCA, which will contribute to the prevention of HSCR.

## 5 CONCLUSION

In conclusion, we suggest that CYP2B6 rs2054675C and rs707265 G alleles are associated with increased susceptibility to the severe HSCR subtype in southern Chinese children. These results indicated different toxic factors from the environment affecting HSCR between eastern and southern Chinese population, which provides a hint of environmental factors for pathogenesis of HSCR. Further study should recruit more TCA patients and include toxic or drug exposure in the association analysis to elucidate environmental factors affecting HSCR.

## CONFLICTS OF INTEREST

The authors declare that there are no conflicts of interest regarding the publication of this paper.

## AUTHORS’ CONTRIBUTIONS

Yun Zhu designed the experiment. Yanqing Liu, Chaoting Lan, Bingxiao Li, Ning Wang, Xiaoyu Zuo, Lihua Huang, and Yuxin Wu collected samples and conducted the study. Yanqing Liu and Yun Zhu analyzed the data. Yanqing Liu and Chaoting Lan wrote the paper. All the authors read and approved the manuscript.

## Data Availability

All of the data used to support the findings of this study are available from the corresponding author upon request.
